# Dermoscopy for the Differentiation of Subacute Cutaneous Lupus Erythematosus from Other Erythematous Desquamative Dermatoses—Psoriasis, Nummular Eczema, Mycosis Fungoides and Pityriasis Rosea

**DOI:** 10.3390/jcm13020577

**Published:** 2024-01-19

**Authors:** Magdalena Żychowska, Kinga Kołcz

**Affiliations:** 1 Department of Dermatology, Institute of Medical Sciences, Medical College of Rzeszow University, 35-959 Rzeszow, Poland; 2 The Doctoral School, University of Rzeszow, 35-959 Rzeszow, Poland

**Keywords:** dermoscopy, videodermoscopy, subacute cutaneous lupus erythematosus, psoriasis, eczema, mycosis fungoides, pityriasis rosea

## Abstract

Subacute cutaneous lupus erythematosus (SCLE) is a condition that might pose a diagnostic challenge. The aim of this study was to assess the usefulness of videodermoscopy in the differentiation of SCLE from other erythematous-desquamative dermatoses. Consecutive patients with SCLE (*n* = 27), psoriasis (*n* = 36), nummular eczema (*n* = 30), mycosis fungoides (*n* = 26), and pityriasis rosea (*n* = 20) referred to our Department of Dermatology were recruited for this study. A representative lesion was visualized using a Canfield D200^EVO^ Videodermatoscope (Canfield Scientific GmbH, Bielefeld, Germany) and evaluated for the following parameters: vessels (morphology and distribution), scales (color and distribution), follicular findings, colors and morphologies, and presence of specific clues. SCLE was predominantly characterized by a polymorphous vascular pattern (92.6%) of unspecific distribution (92.6%) over a pink-red background (74.1%). Gray-brown dots were present in 10 (37.0%) cases, and pigmentation was noted in 15 (55.6%) patients, including peripheral pigmentation in 7 (25.9%) patients. Videodermoscopic evaluation showed significant differences between SCLE and psoriasis, which was characterized by regularly distributed dotted vessels. Although some common dermoscopic features with MF were noted, the presence of yellow structureless areas and red dots/globules favored the diagnosis of MF. In conclusion, a polymorphic vascular pattern, especially in association with gray-brown dots and/or peripheral pigmentation, is a valuable clue for the differentiation of SCLE from other erythematous-desquamative dermatoses.

## 1. Introduction

In recent years, there has been a rapid development of non-invasive skin imaging methods. Dermoscopy, which was initially used mainly for the early detection of skin cancer, has become increasingly utilized in the differential diagnosis of inflammatory skin conditions [1]. The application of dermoscopy in this indication is frequently referred to as inflammoscopy.

Cutaneous lupus erythematosus (CLE), with its three clinical variants—acute CLE (ACLE), subacute CLE (SCLE) and chronic CLE (CCLE)—is characterized by a wide spectrum of dermatological manifestations [2]. SCLE is a highly photosensitive variant of CLE. It is estimated to account for less than 10% of CLE cases and is strongly associated with the presence of anti-Ro autoantibody [3]. SCLE is characterized by symmetrically distributed polycyclic and/or psoriasiform lesions predominantly located on the upper portion of the trunk, upper limbs, and cervical area [3]. Drug-induced SCLE constitutes around one-third of all SCLE cases. It should be suspected particularly in older patients with extensive skin involvement or when bullous and/or targetoid lesions are present [4].

The etiopathogenesis of SCLE is multifactorial and involves a combination of genetic, immunological, and environmental factors [5]. Both innate and cell-mediated immunity have been found to participate in the disease’s development [6]. Histopathologically, SCLE is characterized by moderate hyperkeratosis, liquefactive degeneration of the basal layer, atrophy, and periappendageal infiltration in the superficial dermis composed of mononuclear cells. Dermal edema may be occasionally present. Thickening of the basement membrane and follicular plugs, which constitute a classical feature of DLE, are rarely found in SCLE [7,8]. 

The diagnosis of SCLE is frequently delayed, largely because it might mimic other annular or erythemato-desquamative dermatoses and pose a diagnostic challenge. The psoriasiform variant of SCLE may be confused with psoriasis, and the introduction of phototherapy in this case has significant negative consequences for the patient. Similarly, the early differentiation of SCLE from nummular eczema (NM) or mycosis fungoides (MF) helps direct further diagnostic work-up and enables quick implementation of appropriate treatment. 

Despite the fact that dermoscopy is increasingly used in the differential diagnosis and treatment monitoring of inflammatory skin conditions, limited data are available in the literature on the dermoscopic characteristics of SCLE [9,10,11,12,13,14,15], including, to the best of our knowledge, only two original studies centered around the comparative assessment of dermoscopic findings in specific clinical variants of CLE [14,15]. 

The aim of this study was to assess the usefulness of dermoscopy in the differentiation of SCLE from other erythematous-desquamative dermatoses, e.g., psoriasis, nummular eczema (NM), mycosis fungoides (MF), and pityriasis rosea Gibert. 

## 2. Materials and Methods

Consecutive patients with SCLE and other erythematous-desquamative dermatoses, including psoriasis, NM, patch-stage MF, and pityriasis rosea, referred to our Department of Dermatology over the period of January to October 2023, were recruited for this prospective study. In each case, the diagnosis of SCLE was based on clinical presentation and positive serological findings, as confirmed via histopathology. The diagnosis of MF was histopathologically confirmed in each patient. In the majority of cases of psoriasis and pityriasis rosea, the diagnosis was based on clinical presentation and disease course. In patients with NM, a biopsy was taken to exclude other dermatoses such as MF. 

To avoid the bias associated with anatomical locations, only participants with the aforementioned conditions involving the upper parts of the body (trunk and/or arms) were included. Patients with lesions limited to specific locations such as the face, scalp, palms/soles, intertriginous areas, etc., were not taken into consideration. Patients treated with systemic or topical methods within four weeks prior to referral were excluded from this study. Patients without an established definitive diagnosis were not included either. 

This study was approved by the Bioethics Committee of University of Rzeszow (No. 6/11/2020) and conducted according to the Declaration of Helsinki. All study participants signed written informed consent forms for the use of medical records and publication of images for scientific purposes.

Dermoscopic examinations were performed by the same dermatologist (MŻ) in all cases. The most representative lesion (“target” lesion), preferentially located on the trunk, was selected in each patient. Evaluations were performed using a Canfield D200^EVO^ Videodermatoscope (15–30-fold magnification). For better evaluation of scaling, examination without immersion fluid was performed as the first step. Then, “wet dermoscopy” (with ultrasound gel) was performed for the assessment of capillaries and other structures. During the procedure, minimal pressure was used upon the skin lesions to avoid changes in the vessel morphology.

The images were stored until the time of analysis. Videodermoscopic images were evaluated by two dermatologists for the presence of predefined dermoscopic features [16]. The standardized dermoscopic parameters for assessment in general dermatology, developed by an expert consensus on behalf of the International Dermoscopy Society [16], were used in the current study. These parameters included vessel morphologies (dotted, linear, linear with branches, linear curved), vessel distribution (uniform, clustered, peripheral, reticular, unspecific), scale color (white, yellow, brown), scale distribution (diffuse, central, peripheral, patchy), follicular findings (follicular plugs, follicular red dots, perifollicular white color, perifollicular pigmentation), other structures (presence of colors and/or morphologies such as structureless areas, dots or globules, lines, circles), and specific clues. In addition, several dermoscopic parameters, relevant from the authors’ experience, were added to the analysis. These included polymorphous vessels (at least two vessel morphologies present within the image), vessels distributed in a ring pattern, and scales distributed along the skin lines. Several features, not fitting into the main categories, were classified as specific clues, e.g., “sticky fiber” sign, presence of erosions, or stellate brown structures.

In total, 139 participants were included in this study. This group consisted of 27 patients with SCLE (12 men and 15 women), 36 patients with plaque psoriasis (20 men and 16 women), 30 patients with nummular eczema (21 men and 9 women), 26 patients with MF (14 men and 12 women), and 20 patients with pityriasis rosea (17 men and 3 women). 

The demographic and clinical characteristics of the study population are summarized in Table 1.

### Statistical Analysis

Statistical analysis was performed using SPSS Statistics 29.0. Categorical data are presented as absolute numbers with percentages. Continuous data are presented as mean ± standard deviation (SD) and median with range. To calculate the difference in the frequencies of dermoscopic features in each dermatosis, the Fisher’s exact test was applied.

A *p* value < 0.05 was considered statistically significant. 

The datasets generated during this study are available from the corresponding author upon request.

## 3. Results

### 3.1. Subacute Cutaneous Lupus Erythematosus (SCLE)

**Figure 1:**
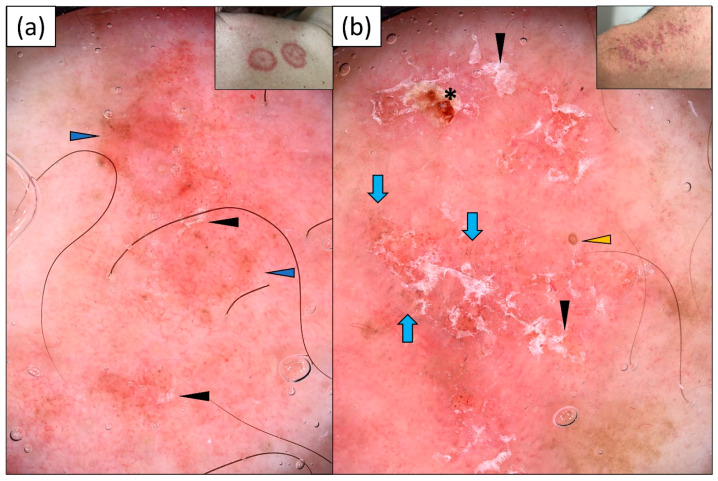
Videodermoscopy of (**a**) annular and (**b**) psoriasiform variants of subacute cutaneous lupus erythematosus (SCLE). (**a**) Polymorphic vascular pattern (dotted and linear vessels) over a pink-red background, fine white scaling (black arrowheads), and peripheral pigmentation (blue arrowheads); (**b**) polymorphic vascular pattern (dotted and linear vessels) over a pink-red background, patchy white scales (black arrowheads), irregularly distributed gray-brown dots (blue arrows), erosion (black asterisk), and a single follicular plug (yellow arrowhead).

SCLE was predominantly characterized by a polymorphous vascular pattern (92.6%) of unspecific distribution (92.6%) over a pink-red background (74.1%). The most frequent vessel morphologies were linear vessels (92.6%), followed by linear branched (63.0%) and dotted vessels (59.3%). White scaling was present in 14 out of 27 patients (51.9%). Of note, gray-brown dots were present in 10 (37.0%) cases, and pigmentation in 15 (55.6%) patients. Peripheral pigmentation was observed in seven (25.9%) patients (Table 2). Sample dermoscopic images are provided in Figure 1.

### 3.2. Psoriasis

**Figure 2:**
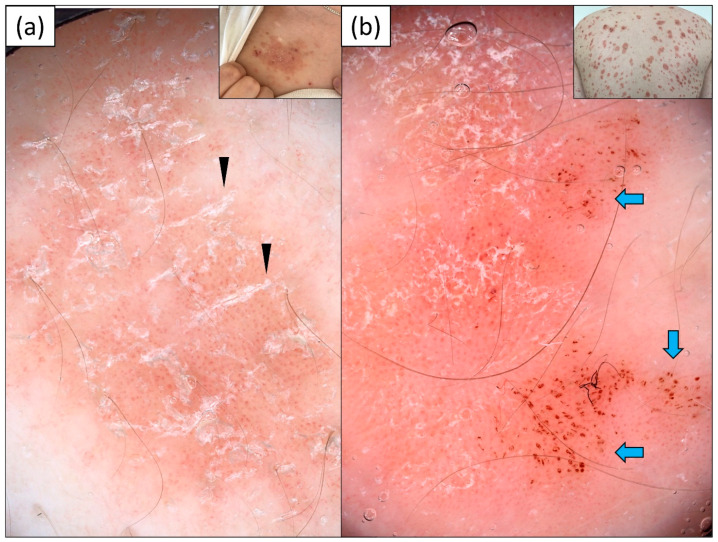
Videodermoscopy of psoriatic plaques: (**a**) regularly distributed dotted vessels on a pink-red background and white scaling arranged linearly along the skin lines (black arrowheads); (**b**) regularly distributed dotted vessels over pink background, fine white scaling, and clustered red hemorrhagic dots and globules (blue arrows).

Dotted vessels were observed in all patients (100%) with psoriasis, and they showed predominantly uniform distribution (72.2%). In single cases, linear and linear curved (hairpin) vessels were noted at the periphery of the psoriatic plaques. White scaling (97.2%) over a pink-red background (74.1%) was present in the vast majority of lesions. However, the distribution of scales was versatile, and included diffuse, peripheral, patchy and, rarely, central arrangements. In four cases (11.1%), the scales were clearly distributed along the skin lines. Of note, there was a high prevalence of red dots (33.3%), red globules (22.2%), and red structureless (hemorrhagic) areas (16.7%) within psoriatic plaques. Details are provided in Table 2. The dermoscopic findings are presented in Figure 2.

### 3.3. Nummular Eczema (NM)

**Figure 3:**
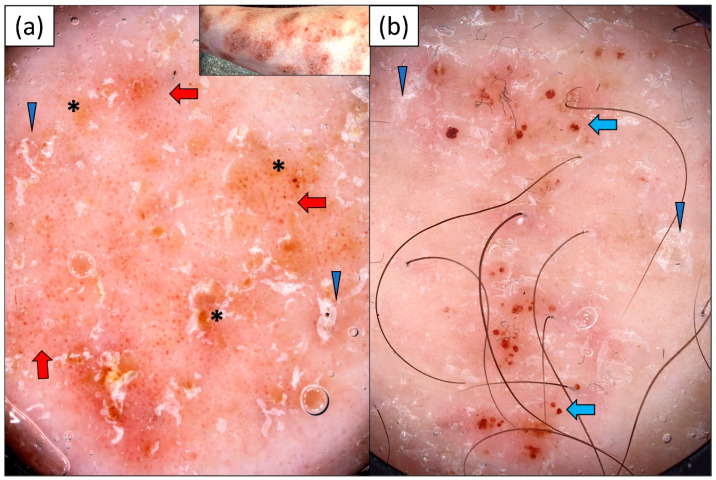
Videodermoscopy of nummular eczema: (**a**) dotted vessels of unspecific, patchy arrangement (red arrows), fine white scaling (blue arrowheads), and focal yellow-orange structureless areas (black asterisk); (**b**) fine white scales (blue arrowhead) over dull pink background and multiple irregularly distributed red hemorrhagic dots and globules of varying sizes (blue arrows).

Dotted vessels (100.0%) were the only vessel morphology observed in NM, and they were predominantly irregularly (non-specifically) distributed (86.7%). The white color of scales (70.0%) prevailed over yellowish scaling (30.0%). Red dots (53.3%), red globules (16.7%), and red hemorrhagic areas (20.0%) were also observed. Other frequent dermoscopic findings in nummular eczema included yellow structureless areas (30.0%), “sticky fiber” sign (23.3%) and erosions (13.3%) (Table 2, Figure 3).

### 3.4. Mycosis Fungoides (MF)

**Figure 4:**
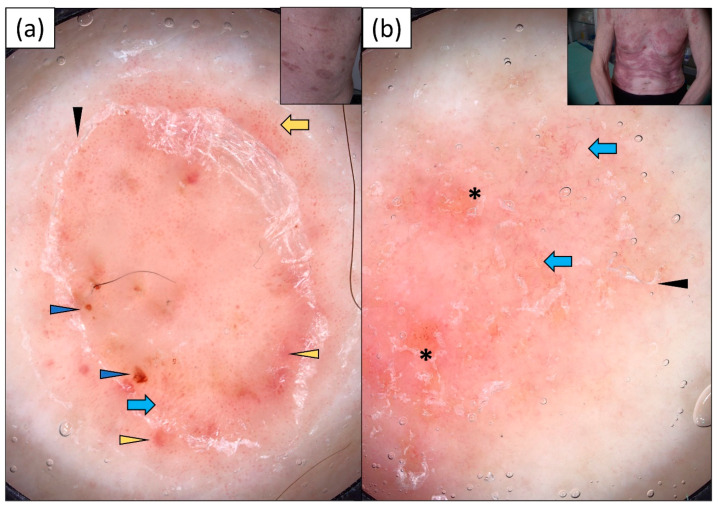
Videodermoscopy of mycosis fungoides: (**a**) irregularly distributed dotted (yellow arrow) and linear curved vessels (blue arrow) over pink background, peripheral whitish scaling (black arrowhead), red hemorrhagic globules (blue arrowhead), and red structureless areas (yellow arrowheads); (**b**) linear and linear curved vessels (blue arrows) of patchy distribution, orange structureless areas (black asterisk), and fine whitish scaling (black arrowhead).

MF was characterized by a wide range of dermoscopic findings. Dotted vessels were the most common vessel morphology, observed in 23 (88.5%) out of 26 cases. They were frequently accompanied by linear, linear branched, or linear curved vessels. Therefore, polymorphous vessels were present in over half of the patients (53.8%). The vascular structures did not show any specific arrangement in the vast majority of cases (84.6%). Both whitish and yellowish scaling was noted in patients with MF, with a predominance of white scales. Other frequent dermoscopic findings included the presence of pigmentation (34.6%), red dots (34.6%), red globules (26.9%), red hemorrhagic areas (23.1%), and gray-brown dots (13.4%). Pink, yellow, or white structureless areas were observed in 23.1%, 19.2%, and 13.4% of cases, respectively (Table 2). Sample dermoscopic images are provided in Figure 4.

### 3.5. Pityriasis Rosea

**Figure 5:**
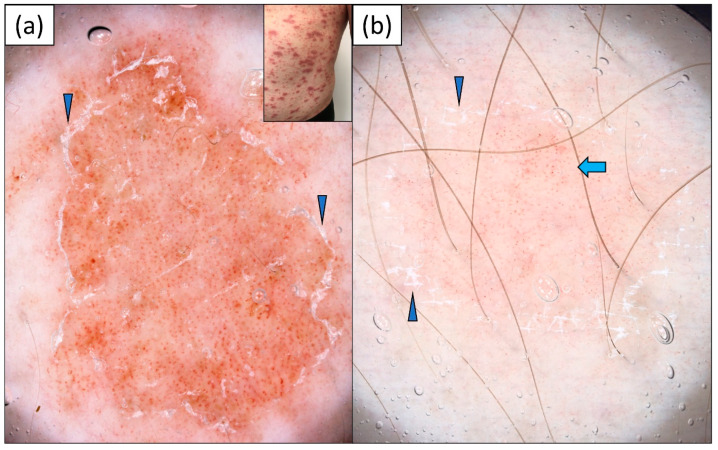
Videodermoscopy of pityriasis rosea Gibert: (**a**) multiple dotted vessels of quite regular distribution over pink-red background and white peripheral (“collarette”) scaling (blue arrowheads); (**b**) dotted vessels in patchy distribution (blue arrow) over dull pink background and peripheral whitish scaling (blue arrowheads) composed of white scaly lines arranged parallel and perpendicular to each other.

Dotted vessels were present in the vast majority of cases (95%) of pityriasis rosea. In five (25.0%) patients, they were associated with linear vessels, and in one (5.0%) patient, with linear curved vessels. White scaling was present in all of the cases, and it was predominantly distributed at the periphery of the lesion (70%). An interesting finding was the presence of white scaling distributed along the skin lines in four (20.0%) patients. In addition, red hemorrhagic dots/globules were present in seven (35.0%) patients (Figure 5). Details are presented in Table 2. 

## 4. Discussion

To date, the dermoscopic features of several erythemato-desquamative dermatoses such as psoriasis [17,18,19,20,21,22] or MF [23,24,25,26] have been elaborated, while there are limited data in the literature on the dermoscopic presentation of SCLE [9,10,11,12,13,14,15]. We performed a comprehensive review of the PubMed database (from database inception to date) for reports of the dermoscopic findings in SCLE. The following keywords were used: “dermoscopy” OR Dermatoscopy” OR “videodermoscopy” OR “videodermatoscopy” combined with “subacute cutaneous lupus erythematosus”. Irrelevant, non-English, and not-full-text papers were excluded from the evaluation. The articles identified during the literature search are summarized in Table 3.

The dermoscopic features of SCLE were initially described by Errichetti et al. [9] based on the analysis of nine serologically and histopathologically confirmed cases. The authors observed the presence of whitish scaling and polymorphic vascular pattern in all patients, which most likely corresponded to hyperkeratosis and vasodilatation, respectively. An additional finding, noted in three cases of SCLE, was the presence of orange-yellowish structureless areas of focal distribution [9]. The biggest study to date, including 30 cases of SCLE, was carried out by Apalla et al. [15]. In that study, the authors compared the dermoscopic findings in SCLE to those of other variants of cutaneous lupus erythematosus (CLE), namely, acute cutaneous lupus erythematosus (ACLE) and chronic cutaneous lupus erythematosus (CCLE). Similar to other reports, they noted a high prevalence of linear curved vessels, predominantly of unspecified (patchy) distribution, and discrete white scaling. The authors also observed white structureless areas, follicular plugs, and orange-yellowish structureless areas in 46.6%, 36.7%, and 30.0% of cases, respectively. 

There is also one case report in the literature presenting the potential application of dermoscopy for treatment monitoring in SCLE [12]. The authors suggested that the disappearance of scales, follicular plugs, and shiny white structures (visible only under polarized dermoscopy), as well as the reduction in vascular structures, may be indicators of good treatment response. However, it should be taken into account that larger studies are needed to draw reliable conclusions. 

In the current study, we provide new insights into the dermoscopic findings of SCLE. The recognition of the dermoscopic pattern of each of the erythematous-desquamative conditions may facilitate the primary diagnosis and direct further management. 

The results of the current study are consistent with the limited literature data on the dermoscopic presentation of SCLE that are available so far. SCLE was predominantly characterized by polymorphous vessels of unspecific distribution and patchy white scales. Interestingly, we observed a high prevalence of gray-brown dots (10/27 cases; 37.0%) in patients with SCLE. They were more frequently present in SCLE than in the other erythematous-scaly dermatoses (*p* < 0.01). Gray color in dermoscopy has been linked to the presence of melanophages in histopathological examination [27]. Gray-brown dots or peppering have already been reported in SCLE, both in patients of low skin phototypes [14,15] and in dark-skinned individuals [11,12]. In the study by Apalla et al. [15], gray-brown dots were noted in SCLE and CCLE but not in ACLE. However, their presence (or absence) alone was not sufficient to distinguish between the different variants of cutaneous lupus. Our results imply that the presence of gray-brown dots may be particularly helpful in differentiating between SCLE and common mimickers, namely, psoriasis and eczema. In the two latter conditions, we have not observed any case showing gray structures under dermoscopy. 

The presence of peripheral pigmentation might also constitute a valuable clue for the diagnosis of SCLE. In our study, peripheral pigmentation was noted in seven patients (25.9%) with SCLE and in only a single case (3.8%) of MF. In addition, we observed the presence of a peculiar pigmentation in the form of irregular brown lines meeting at a common point in three (11.1%) patients with SCLE. We referred to this dermoscopic finding as “stellate brown structures”. Their presence was not noted in any of the other analyzed dermatoses.

Follicular keratotic plugs are a dermoscopic phenomenon regularly observed in discoid lupus erythematosus (DLE), the most common variant of CCLE [28]. Histologically, they are linked to the follicular hyperkeratosis and plugging of the follicular ostia [29]. They were reported both in scalp and non-scalp DLE lesions with a mean frequency of 47% and 66.7% of cases, respectively [28]. The possible presence of keratotic plugs in SCLE has been pointed out in previous publications [11,14,15]. In the current study, they were observed in 5 out of 27 (18.5%) cases of SCLE. It is worth noting that the presence of follicular keratotic plugs does not allow for a definite differentiation between SCLE and CCLE/DLE. 

The presence of yellow-orange structureless areas, focally distributed within SCLE lesions, was initially reported by Errichetti et al. [9]. Orange structureless areas were also observed in a subsequent study by Apalla et al. [15] in 9 out of 30 (30.0%) SCLE cases, in 12 out of 44 (27.3%) CCLE cases, and 2 out of 5 (40.0%) ACLE cases. The orange color in dermoscopy was suggested to correlate with dense lymphocytic infiltration in histopathology. However, such correlation in CLE has not yet been definitely confirmed [15]. Interestingly, we did not note yellow/orange areas in any patient in our SCLE group. On the contrary, yellow structureless areas were predominantly present in NM (30.0%), followed by MF (19.2%). In these cases, they most probably corresponded to spongiosis and serous exudate. 

Although SCLE may clinically resemble psoriasis in many cases, it appears that, dermoscopically, the two diseases differ significantly. In accordance with the literature data, psoriatic plaques showed regularly distributed dotted vessels and whitish scaling in the vast majority of cases [17,18,19,20]. Taking this into account, the vessel morphology and their distribution should be a significant help in differentiating between the psoriasiform variants of SCLE and psoriasis. In addition, the presence of red dots or globules favors the diagnosis of psoriasis, while brown-gray dots are an indicator of SCLE. 

Interestingly, under dermoscopy, SCLE showed several similarities to MF. In both conditions, polymorphous vessels of unspecified distribution were predominant. The major difference was, firstly, the presence of yellow structureless areas in MF that were not noted in SCLE. Secondly, the presence of red dots/globules favored the diagnosis of MF, while gray-brown dots were more frequently observed in MF. 

An advantage of the current study is the use of videodermoscopy for the assessment of skin lesions. Contrary to handheld dermatoscopes, which offer 10-fold magnification, videodermatoscopes enable more detailed analysis of the structures, including vessel morphologies, and storage of good-quality images. The use of higher magnifications most likely made it easier for us to observe the gray-brown dots in SCLE. We postulate that some of these cases would have been missed if the examination had been performed only with a hand-held device. 

The limitations of this study include its single-center design. In addition, we did not directly correlate the dermoscopic findings with histopathological features. This study was carried out among patients with fair skin phototypes; therefore the result might not be attributable to the skin of people of color. 

## 5. Conclusions

Dermoscopy might be of help in differentiating SCLE from other erythematous-desquamative dermatoses such as psoriasis, eczema, MF, or pityriasis rosea. A polymorphic vascular pattern, especially in association with gray-brown dots and/or peripheral pigmentation, is a valuable clue for the diagnosis of SCLE. 

## Figures and Tables

**Table 1:** Demographic and clinical characteristics of study participants (SCLE—subacute cutaneous lupus erythematosus; MF—mycosis fungoides; *n*—number of cases).

Clinical Characteristics	Diagnosis				
	SCLE *n* = 27	Psoriasis *n* = 36	Nummular Eczema *n* = 30	MF *n* = 26	Pityriasis Rosea *n* = 20
Sex, *n* (%)					
Male	12	20	21	14	17
Female	15	16	9	12	3
Age, years					
Mean ± SD	65.2 ± 12.3	49.1 ± 19.2	42.5 ± 17.8	69.5 ± 11.7	49.1 ± 11.9
Median (range)	71.0	49.0	42.5	71.0	50.0
Fitzpatrick skin phototype, *n* (%)					
I	10	8	5	9	3
II	17	25	21	16	16
III	0	3	4	1	1

**Table 2:** Dermoscopic findings in study participants (SCLE—subacute cutaneous lupus erythematosus; MF—mycosis fungoides; *n*—number of cases.

Dermoscopic Characteristics	SCLE *n* = 27	Psoriasis *n* = 36	Nummular Eczema *n* = 30	MF *n* = 26	Pityriasis Rosea *n* = 20	*p* Value	Specificity	PPV
**Morphology of vessels, *n* (%)**								
Dotted	16 (59.3)	36 (100.0)	30 (100.0)	23 (88.5)	19 (95.0)	<0.01	3.9%	12.9%
Linear	25 (92.6)	1 (2.8)	0 (0.0)	10 (38.5)	5 (25.0)	<0.01	85.7%	61.0%
Linear with branches	17 (63.0)	0 (0.0)	0 (0.0)	4 (13.4)	0 (0.0)	<0.01	96.4%	81.0%
Thick	4 (14.8)	0 (0.0)	0 (0.0)	1 (3.8)	0 (0.0)	0.01	99.1%	80.0%
Thin	16 (59.3)	0 (0.0)	0 (0.0)	4 (13.4)	0 (0.0)	<0.01	96.4%	80.0%
Linear curved	2 (7.4)	2 (5.6)	0 (0.0)	9 (34.6)	1 (5.0)	<0.01	89.3%	14.3%
Polymorphous	25 (92.6)	3 (8.3)	0 (0.0)	14 (53.8)	5 (25.0)	<0.01	80.4%	53.2%
**Distribution of vessels, *n* (%)**								
Uniform	0 (0.0)	26 (72.2)	2 (6.7)	0 (0.0)	9 (45.0)	<0.01	67.0%	0.0%
Clustered	0 (0.0)	1 (2.8)	2 (6.7)	0 (0.0)	0 (0.0)	0.47	97.3%	0.0%
Peripheral	2 (7.4)	4 (11.1)	0 (0.0)	2 (7.7)	1 (5.0)	0.44	93.8%	22.2%
Unspecific	25 (92.6)	5 (13.9)	26 (86.7)	22 (84.6)	8 (40.0)	<0.01	45.5%	29.1%
Ring pattern	0 (0.0)	3 (8.3)	0 (0.0)	0 (0.0)	0 (0.0)	0.24	97.3%	0.0%
**Color of scales, *n* (%)**								
White	14 (51.9)	35 (97.2)	21 (70.0)	19 (73.1)	20 (100.0)	<0.01	15.2%	12.8%
Yellow	1 (3.7)	0 (0.0)	9 (30.0)	3 (11.5)	0 (0.0)	<0.01	89.3%	7.7%
**Distribution of scales, *n* (%)**								
Diffuse	1 (3.7)	10 (27.8)	8 (26.7)	7 (26.9)	0 (0.0)	<0.01	77.7%	3.8%
Central	2 (7.4)	3 (8.3)	0 (0.0)	0 (0.0)	0 (0.0)	0.19	97.3%	40.0%
Peripheral	4 (14.8)	14 (38.9)	3 (10.0)	5 (19.2)	14 (70.0)	<0.01	67.9%	10.0%
Patchy	7 (25.9)	13 (36.1)	11 (36.7)	12 (46.2)	7 (35.0)	0.67	61.6%	14.0%
Along skin lines	0 (0.0)	4 (11.1)	0 (0.0)	1 (3.8)	4 (20.0)	0.01	92.0%	0.0%
**Follicular findings, *n* (%)**								
Follicular plugs	5 (18.5)	1 (2.8)	1 (3.3)	2 (7.7)	0 (0.0)	0.08	96.4%	55.6%
Perifollicular white halo	0 (0.0)	0 (0.0)	0 (0.0)	2 (7.7)	0 (0.0)	0.28	98.2%	0.0%
Perifollicular scaling	0 (0.0)	0 (0.0)	0 (0.0)	1 (3.8)	2 (10.0)	0.04	97.3%	0.0%
**Morphologies/colors, *n* (%)**								
White structureless areas	1 (3.7)	0 (0.0)	0 (0.0)	4 (13.4)	0 (0.0)	<0.01	96.4%	20.0%
Pink structureless areas	4 (14.8)	0 (0.0)	1 (3.3)	6 (23.1)	0 (0.0)	<0.01	93.8%	36.4%
Yellow structureless areas	0 (0.0)	1 (2.8)	9 (30.0)	5 (19.2)	0 (0.0)	<0.01	86.6%	0.0%
Orange structureless areas	0 (0.0)	1 (2.8)	1 (3.3)	0 (0.0)	0 (0.0)	1.00	98.2%	0.0%
Dots/globules	10 (37.0)	14 (38.9)	22 (73.3)	14 (53.8)	7 (35.0)	0.02	49.1%	14.9%
Red dots	1 (3.7)	12 (33.3)	16 (53.3)	9 (34.6)	6 (30.0)	<0.01	61.6%	2.3%
Red globules	1 (3.7)	8 (22.2)	5 (16.7)	7 (26.9)	2 (10.0)	0.13	80.4%	4.3%
Gray-brown dots	10 (37.0)	0 (0.0)	0 (0.0)	4 (13.4)	1 (5.0)	<0.01	95.5%	66.7%
Gray-brown globules	0 (0.0)	0 (0.0)	0 (0.0)	1 (3.8)	0 (0.0)	0.33	99.1%	0.0%
White-yellowish globules	0 (0.0)	1 (2.8)	1 (3.3)	0 (0.0)	0 (0.0)	1.00	98.2%	0.0%
White lines	0 (0.0)	0 (0.0)	0 (0.0)	1 (3.8)	0 (0.0)	0.33	99.1%	0.0%
Circles	0 (0.0)	0 (0.0)	0 (0.0)	1 (3.8)	0 (0.0)	0.33	99.1%	0.0%
**Specific clues, *n* (%)**								
Yellowish crust	0 (0.0)	0 (0.0)	2 (6.7)	0 (0.0)	0 (0.0)	0.14	98.2%	0.0%
Erosion	1 (3.7)	0 (0.0)	4 (13.3)	0 (0.0)	0 (0.0)	0.03	96.4%	20.0%
“Sticky fiber” sign	0 (0.0)	0 (0.0)	7 (23.3)	0 (0.0)	0 (0.0)	<0.01	93.8%	0.0%
Dull pink background	6 (22.2)	6 (16.7)	18 (60.0)	15 (57.7)	6 (30.0)	<0.01	59.8%	11.8%
Pink-red background	20 (74.1)	30 (83.3)	12 (40.0)	11 (42.3)	13 (65.0)	<0.01	41.7%	23.3%
Red hemorrhagic areas	2 (7.4)	6 (16.7)	6 (20.0)	6 (23.1)	1 (5.0)	0.33	83.0%	9.5%
Pigmentation	15 (55.6)	2 (5.6)	5 (16.7)	9 (34.6)	1 (5.0)	<0.01	84.8%	46.9%
Peripheral pigmentation	7 (25.9)	0 (0.0)	0 (0.0)	1 (3.8)	0 (0.0)	<0.01	99.1%	87.5%
Yellow-orange round structures	0 (0.0)	0 (0.0)	1 (3.3)	1 (3.8)	0 (0.0)	0.60	98.2%	0.0%
White follicular dots	2 (7.4)	0 (0.0)	0 (0.0)	0 (0.0)	0 (0.0)	0.09	100.0%	100.0%
Stellate brown structures	3 (11.1)	0 (0.0)	0 (0.0)	0 (0.0)	0 (0.0)	0.02	100.0%	100.0%

**Table 3:** Summary of the available literature on the dermoscopic presentation of subacute cutaneous lupus erythematosus (SCLE). N/A—not available.

Author, Year	Type of Study	Number of Cases	Type of Dermatoscope, Magnification	Main Dermoscopic Findings					
				Vessels	Scales		Follicular Findings	Morphologies/Colors	Remarks
					Color	Distribution			
Errichetti et al., 2016 [9]	Case series	9	Handheld DermLite DL3, ×10	Linear (4/9) Linear irregular (6/9) Sparsely distributed dotted (8/9) Branched (3/9)	White (100%; 9/9)	Peripheral (5/9) Diffuse (4/9)		Focal orange-yellowish structureless areas (3/9)	
Apalla et al., 2020 [15]	Original	30	DermLite Photosystem, ×10	Linear curved (10/30) Dotted (10/30) Linear branched (5/30) Linear (2/30)	White (21/30) Yellow-brown (11/30)	Patchy (16/30) Diffuse (5/30)	Follicular plugs/rosettes (11/30) Perifollicular white halo (7/30)	Erythema (26/30) Pink structureless areas (26/30) White structureless areas (14/30) Orange structureless areas (9/30) Brown structureless areas (1/30) Gray-brown dots (2/30) Erosion (7/30)	
Behera et al., 2021 [12]	Case report	1	DermLite DL4, ×10	Linear (1/1) Linear curved/comma (1/1) Focal dotted (1/1)	White (1/1)	Patchy (1/1) Diffuse (1/1)	Follicular plugs (1/1)	White structureless area (1/1) White shiny structures (1/1) Focal multicolored pattern (1/1) Brown to blue-gray peppering (1/1)	Treatment follow-up by dermoscopy
Behera et al., 2021 [11]	Case report	1	Heine Delta 20, ×10	Linear (1/1) Hairpin (1/1) Glomerular (1/1)	White (1/1)	N/A	none	White to reddish-white homogenous area (1/1) Brown to blue-gray dots (1/1) Blue-gray peppering (1/1) White homogenous area around blood vessel (1/1)	
Mazzilli et al., 2021 [10]	Case series	2	N/A	Mixed vascular pattern (2/2)	White (1/1)	Diffuse (1/1)	none	”pigment areas” (1/1) Focal yellowish areas (1/1)	Additional report of confocal findings
Montero-Menarguez et al., 2022 [13]	Case report	1	N/A	Thin arborizing (1/1)	White (1/1)	N/A	Big yellow dots (1/1)	Erythema (1/1)	Additional report of confocal findings
Żychowska et al., 2022 [14]	Original	11	Canfield D200^EVO^ videodermatoscope (×20–70)	Linear (8/11) Linear branched (8/11) Linear curved (8/11) Dotted (7/11)	White (7/11) Yellow (1/11)	Patchy (8/11) Peripheral (1/11)	Follicular plugs (2/11)	Pink-red background (10/11) Peripheral pigmentation (5/11) Red hemorrhagic areas (4/11) Dilated follicles (3/11) Gray-brown dots (2/11) Erosion (2/11) White-yellowish globules (1/11) Pink structureless areas (1/11) ”sticky fiber” sign (1/11)	

## Data Availability

Data are available from corresponding author upon reasonable interest.
